# Effect of pubertal induction with combined gonadotropin therapy on testes development and spermatogenesis in males with gonadotropin deficiency: a cohort study

**DOI:** 10.1093/hropen/hoaf026

**Published:** 2025-05-13

**Authors:** Sebastian Castro, Kyla Ng Yin, Francesco d’Aniello, Emma C Alexander, Emily Connolly, Claire Hughes, Lee Martin, Rathi Prasad, Helen L Storr, Ruben H Willemsen, Leo Dunkel, Gary Butler, Sasha R Howard

**Affiliations:** Centre for Endocrinology, William Harvey Research Institute, Barts and the London School of Medicine and Dentistry, Queen Mary University of London, London, UK; Centro de Investigaciones Endocrinológicas “Dr. César Bergadá” (CEDIE), CONICET—FEI—División de Endocrinología, Hospital de Niños Ricardo Gutiérrez, Buenos Aires, Argentina; Centre for Endocrinology, William Harvey Research Institute, Barts and the London School of Medicine and Dentistry, Queen Mary University of London, London, UK; Centre for Endocrinology, William Harvey Research Institute, Barts and the London School of Medicine and Dentistry, Queen Mary University of London, London, UK; School of Pediatrics, University of Rome Tor Vergata, Rome, Italy; Endocrinology and Diabetes Unit, Bambino Gesù Children’s Hospital, IRCCS, Rome, Italy; Centre for Endocrinology, William Harvey Research Institute, Barts and the London School of Medicine and Dentistry, Queen Mary University of London, London, UK; Paediatric Intensive Care Unit, St Mary’s Hospital, Imperial College Healthcare NHS Trust, London, UK; Department of Paediatric Endocrinology, Royal London Children’s Hospital, Barts Health NHS Trust, London, UK; Department of Paediatric Endocrinology, Royal London Children’s Hospital, Barts Health NHS Trust, London, UK; Department of Paediatric Endocrinology, Royal London Children’s Hospital, Barts Health NHS Trust, London, UK; Centre for Endocrinology, William Harvey Research Institute, Barts and the London School of Medicine and Dentistry, Queen Mary University of London, London, UK; Department of Paediatric Endocrinology, Royal London Children’s Hospital, Barts Health NHS Trust, London, UK; Centre for Endocrinology, William Harvey Research Institute, Barts and the London School of Medicine and Dentistry, Queen Mary University of London, London, UK; Department of Paediatric Endocrinology, Royal London Children’s Hospital, Barts Health NHS Trust, London, UK; Department of Paediatric Endocrinology, Royal London Children’s Hospital, Barts Health NHS Trust, London, UK; Centre for Endocrinology, William Harvey Research Institute, Barts and the London School of Medicine and Dentistry, Queen Mary University of London, London, UK; Department of Paediatric and Adolescent Endocrinology, University College London Hospital NHS Foundation Trust, London, UK; UCL GOS Institute of Child Health, University College London, London, UK; Centre for Endocrinology, William Harvey Research Institute, Barts and the London School of Medicine and Dentistry, Queen Mary University of London, London, UK; Department of Paediatric Endocrinology, Royal London Children’s Hospital, Barts Health NHS Trust, London, UK

**Keywords:** puberty, fertility, spermatogenesis, gonadotropins, hypogonadotropic hypogonadism

## Abstract

**STUDY QUESTION:**

Are recombinant FSH (rFSH) and hCG effective therapies for promoting testicular growth and spermatogenesis in male adolescents and young adults with gonadotropin deficiency?

**SUMMARY ANSWER:**

Combined gonadotropin therapy is effective in inducing puberty and promoting spermatogenesis in male adolescents and young adults with gonadotropin deficiency and has the potential to improve adult outcomes relating to both fertility and quality of life.

**WHAT IS KNOWN ALREADY:**

Deficiency of pituitary gonadotropins (LH and FSH) due to hypogonadotropic hypogonadism (HH) can result in poor testicular development, low testicular volumes, micropenis and cryptorchidism. Inadequate hormonal replacement can lead to long-term issues, including subfertility or infertility, and reduced quality of life. Exogenous testosterone for pubertal induction can elevate serum testosterone concentrations and induce virilization, but it does not promote testicular development nor induce spermatogenesis. Fertility and testes growth remain primary concerns for patients seeking treatment.

**STUDY DESIGN, SIZE, DURATION:**

We conducted a retrospective observational review of male adolescents and young adults with gonadotropin deficiency and seeking puberty replacement therapy at two large tertiary centre hospitals in London, UK, from 2010 to 2024.

**PARTICIPANTS/MATERIALS, SETTING, METHODS:**

A total of 35 males, with diagnosis of congenital hypogonadotropic hypogonadism (CHH: n = 23; further subdivided into those with partial [pHH: n = 8] and those with complete gonadotropin deficiency [cHH: n = 15]), acquired HH (AHH: n = 4) or Kallmann syndrome (KS: n = 8), received combined gonadotropin therapy. We assessed testicular growth and semen quality post-treatment.

**MAIN RESULTS AND THE ROLE OF CHANCE:**

The majority of patients were referred for pubertal delay, alone or in combination with cryptorchidism, micropenis or microorchidism. Out of 35 patients, 22 (63%) had previously received testosterone, and the median age at gonadotropin treatment initiation was 15.8 years (range: 11.8–22.7). Semen analysis was obtained in 18 out of 19 patients who had received gonadotropin therapy for a median treatment duration of 21.1 months (range: 4.5–66.9) for rFSH and 19.5 months (range: 8.3–61.1) for hCG. The median sperm count on semen analysis was 8.9 × 10^6^/ml (range: 0.0–54.9). Significant increases were noted in testicular volume (median change after therapy: 10.5 ml [95% CI 9.5–13.6], *P* < 0.0001), testosterone (median increase: 25.7 nmol/l [95% CI 19.8–31.5], *P* < 0.0001) and inhibin B levels (67.7 pg/ml [95% CI 18.4–86.7], *P* = 0.0008).

**LIMITATIONS, REASONS FOR CAUTION:**

The relatively low representation of patients with acquired HH in our study emphasizes the need to extrapolate the findings with caution in this specific subgroup of adolescent males with HH. The study is also an observational one, therefore meaning that some outcomes (such as change in inhibin B concentration) were not collected routinely and not reported for all patients. The observational nature of the study design also accounts for the differences in doses and duration observed in gonadotropin therapy.

**WIDER IMPLICATIONS OF THE FINDINGS:**

The treatment of adult male infertility is particularly difficult in severe forms of gonadotropin deficiency, where there has been no testicular stimulation during mini-puberty or puberty. Appropriate hormonal replacement in puberty with combined gonadotropins can induce testicular maturation and spermatogenesis, but data are limited and at present, there is no international consensus on best practice regimens in adolescent and young adult males. Our treatment protocol induced testicular growth and caused increases in serum testosterone and Sertoli cell biomarkers, and spermatogenesis in 15/18 of patients who had completed semen analysis. This indicates the potential to substantially improve the reproductive, physical, and psychological health of patients who have a significant and unmet need for adequate hormonal replacement during puberty. The study described here included patients with diverse forms of HH (congenital, acquired, complete, and partial HH), thereby providing encouraging results across a variety of subjects with impaired puberty facing increased odds of fertility problems in adulthood. Additionally, we observed similar sperm counts between those who received exogenous testosterone treatment prior to gonadotropin therapy and those who began directly on gonadotropins for pubertal induction. This last finding is aligned with previous data and may help to reassure paediatric endocrinologists with limited access to rFSH or hCG that the use of exogenous testosterone to induce androgen-dependent changes in patients seeking treatment for pubertal delay is unlikely to compromise spermatogenic potential, should gonadotropins become available at a later stage.

**STUDY FUNDING/COMPETING INTEREST(S):**

S.C. was funded by an ESPE Early Career Scientific Development Grant. S.R.H. was funded by the Wellcome Trust (222049/Z/20/Z) and Barts Charity [MGU0552]. K.N.Y. was employed under the NIHR Specialist Foundation Programme. F.d.A. was funded by the student traineeship, University of Rome ‘Tor Vergata’, an Erasmus Grant and an ESPE Early Career Scientific Development Grant. E.C.A. was funded by an NIHR Academic Clinical Fellowship (ACF-2021-19-002). The views expressed in this publication are those of the author(s) and not necessarily those of the NIHR, NHS, or the UK Department of Health and Social Care. G.B. received an ESPE Mid-Career Research Fellowship to enable the development of the clinical treatment schedule. The authors have no conflicting interests.

**TRIAL REGISTRATION NUMBER:**

N/A.

WHAT DOES THIS MEAN FOR PATIENTS? The management of male infertility is very challenging, particularly in severe forms of hormone deficiency. These hormones are specific signals produced by the brain, which direct development of the testicles or ovaries. Patients who are unable to produce these signals (named ‘gonadotropin hormones’) have a condition called gonadotropin deficiency (GD).

For men with GD, medical therapy for fertility is often unsuccessful. This is because hormones drive the development of the testicles in infancy and at puberty, a vital process to enable later sperm production. Despite adult hormone replacement, individuals may suffer infertility in addition to chronic health and psychological issues. People born with a congenital cause for GD (due to changes in their genes) are at the highest risk.

Standard treatment for lack of puberty in adolescent and young adult males with GD is testosterone replacement. While testosterone helps with physical development, it does not make testicles grow and make sperm. Thus, the potential to father a child and to complete pubertal development are still of major concern for patients.

This study looks at whether treatment with gonadotropin hormones instead of testosterone can lead to growth of the testicles and enable sperm production in males with GD. We found that testicular volumes after gonadotropin treatment were, on average, comparable to those of healthy adult men (15 ml), and that more than 80% of those who completed therapy succeeded in producing good-quality sperm.

We concluded that gonadotropin therapy is effective in promoting the growth of the testicles and allowing sperm production in male adolescents and young adults with this condition, suggesting the potential to facilitate fatherhood.

## Introduction

Hypogonadotropic hypogonadism (HH) usually results from an insufficient secretion of gonadotropin-releasing hormone (GnRH) from the mediobasal hypothalamus, resulting in deficiency of gonadotropin secretion (LH and FSH), by the anterior pituitary gland, and consequently decreased endogenous sex steroid production by the gonads (Dodé and Hardelin, 2009; Argente *et al.*, 2023). HH may be either congenital (CHH), usually due to genetic conditions affecting GnRH or gonadotropin secretion, or acquired (AHH), due to direct damage to the hypothalamus or pituitary. HH typically manifests as absent, partial or arrested pubertal development during adolescence (Salenave *et al.*, 2012; Varimo *et al.*, 2017).

In males, the hypothalamic–pituitary–gonadal axis is typically active from the prenatal period through the first six months of post-natal life, during a phase known as ‘mini-puberty’. This activation results in an increase in LH and FSH and subsequently in the sex steroids produced by the testes, leading to early penile and testicular growth (Rey *et al.*, 2013; Nordenström, 2022; Rohayem *et al.*, 2024). In healthy peripubertal males, FSH promotes the proliferation of testicular Sertoli cells, thereby facilitating testicular growth and enabling post-pubertal spermatogenesis. However, during mini-puberty, Sertoli cells do not express androgen receptors, preventing the onset of spermatogenesis during infancy (Cortes *et al.*, 1987; Chemes *et al.*, 2008).

In male patients with CHH, the lack of gonadotropin stimulation during mini-puberty and subsequent adolescence impedes testicular maturation, often resulting in low testicular volumes, micropenis and/or cryptorchidism (undescended testis/es). As FSH upregulates inhibin B and anti-Müllerian hormone (AMH) synthesis by Sertoli cells, these serve as crucial biomarkers for diagnosing CHH and monitoring the therapeutic response to gonadotropin treatment for the induction of puberty and spermatogenesis (Rohayem *et al.*, 2015). The natural history of untreated CHH and AHH includes broader consequences in later life, such as low testicular volumes, diminished sexual function, compromised bone health, psychological distress, reduced quality of life, and ultimately infertility (Rohayem *et al.*, 2015; Dwyer *et al.*, 2019; Young *et al.*, 2019; Nordenström *et al.*, 2022).

These outcomes underscore the clinical rationale for initiating the induction of secondary sexual characteristics in male patients with HH, with current practice most commonly initiating exogenous testosterone from 12 years of age (Federici *et al.*, 2022; Nordenström *et al.*, 2022). However, while exogenous testosterone can elevate serum testosterone concentrations and induce virilization, it does not promote testicular development nor induce spermatogenesis in adolescent and young adult males with this condition. Fertility remains a primary concern for patients seeking treatment (Rohayem *et al.*, 2017).

Consequently, there has been growing interest in the use of gonadotropins for pubertal induction, as this approach not only results in physical growth, development of secondary sexual characteristics, and penile growth, but also promotes testicular growth and maturation with the potential for spermatogenesis due to endogenous testosterone production (Tsai-Morris *et al.*, 2010).

However, data on paediatric cohorts are limited. Well-designed retrospective studies provide essential real-world evidence that can guide clinical practice. As randomized controlled trials (RCTs) are often unfeasible in rare diseases such as HH due to limited patient numbers and logistical constraints, these studies play a critical role in advancing the field. A recent systematic review and metanalysis investigating the efficacy of gonadotropins for pubertal induction in males with HH concluded that the combination of hCG with recombinant (r) FSH induces significant increases in penis size, testosterone and inhibin B serum concentrations (Alexander *et al.*, 2024). When compared to hCG monotherapy or to other therapeutic regimens, combined hCG/rFSH treatment promoted greater testicular development and higher rates of spermatogenesis. This review included the only multicentre prospective randomized controlled trial to date in adolescents, which supported the use of combined hCG/rFSH treatment in pubertal induction in patients with CHH (Rohayem *et al.*, 2017).

In patients with severe CHH, pre-treatment with rFSH aims to emulate the infantile mini-puberty period of Sertoli cell stimulation, in order to optimize response to combined therapy. A randomized open-label trial by Dwyer *et al.* (2013) evaluated the efficacy of rFSH pre-treatment for inducing testicular growth and fertility in adult men with HH with pre-pubertal testes, concluding that rFSH pre-treatment optimizes the Sertoli cell population and stimulates morphological changes to the testicular architecture. A retrospective clinical study by Raivio *et al.* (2007) showed similar findings when pre-treatment with rFSH was used in boys with pre-pubertal onset of HH. On the other hand, due to milder phenotype, several reports demonstrated better fertility outcomes in males with AHH compared to those with CHH (Mao *et al.*, 2015; Dwyer, 2022; Maione *et al.*, 2022).

HH is one of the rare endocrine diseases where medical therapy has the potential to radically improve physiological function (Kumar *et al.*, 2006). However, at present there is no standardized approach to this treatment in adolescent males with no national or international guidelines. A recently published systematic review concluded that men with CHH are likely to achieve spermatogenesis after treatment with gonadotropins or pulsatile GnRH, although their sperm quality remains lower than that of healthy men. This underscores the need for evidence-based data on protocols and guidelines for the optimal treatment approach for males with CHH (Dwyer *et al.*, 2024). Our tertiary centres in London, UK have previously developed consensus guidelines for the use of combined gonadotropins, with pre-treatment with rFSH for those with severe CHH. Through a retrospective analysis of patients who were treated based on these harmonized guidelines (Dunkel *et al.*, 2021), we describe responses to therapy in a cohort of males with a broad spectrum of aetiologies of HH, who received recombinant gonadotropin therapy for the induction or completion of puberty, with the aim of assessing efficacy and safety of these therapies for outcomes of testicular growth and semen quality.

## Materials and methods

### Therapeutic design and setting

This was a retrospective, observational, review of male adolescents with HH receiving treatment for pubertal induction. Participants were included from paediatric referral endocrinology outpatient clinics in two large tertiary centre hospitals in London, UK: Royal London Hospital, Barts Health (RLH) and University College London Hospital (UCLH), from January 2010 to July 2024. We aimed to describe semen production and quality, and the change in testicular volume (TV) after completing therapy with recombinant gonadotropins. For assessing changes in both TV and serum concentrations of FSH, testosterone, AMH, and inhibin B, we used a pre-/post-study design.

### Ethical issues

Ethical approval was granted by the London–Chelsea NRES committee and the UK NHS Health Research Authority (13/LO/0257), with all parents or guardians of child patients providing written informed consent, and adolescents providing their own written informed assent.

### Participants and data gathering methods

#### Inclusion criteria

All male patients with a clinical and biochemical diagnosis of hypogonadotropic hypogonadism, of congenital or acquired aetiology, and requiring treatment to induce or complete puberty, were offered this treatment regimen. Genetic testing was used to support the diagnosis of congenital conditions via NHS gene panel testing (Panelapp, 2023a, https://panelapp.genomicsengland.co.uk/panels/650/; Panelapp, 2023b, https://panelapp.genomicsengland.co.uk/panels/483/).

#### Exclusion criteria

Patients with functional hypogonadism due to low caloric intake, excessive exercise or chronic disease, primary hypogonadism, or constitutional or self-limited delayed puberty were excluded. Data from subjects not treated with gonadotropins or receiving a course of recombinant gonadotropin therapy for less than 6 months, thus insufficient for semen sampling or assessing changes in TV, were also excluded.

#### Laboratory test methods

LH and FSH were determined using electro-chemiluminescent immunoassays ECLIA (Roche, Mannheim, Germany). The limits of quantification of both LH and FSH assays were 0.10 IU/l; intra- and inter-assay coefficients of variation were 1.1–1.4% and 1.5–1.8%, respectively for LH, and 1.0–1.1% and 4.1–4.2% for FSH. The limit of quantification was 0.1 IU/l. Inhibin B was measured using enzyme-linked immunoassay (ELISA, Biomatik, Wilmington, DE, USA); intra- and inter-assay coefficients of variation were 3.5–5.6% and 6.2–7.6%, respectively and the lower limit of detection was 9.8 pg/ml. AMH was determined using an ELISA (Beckman-Coulter, Brea, CA, USA); intra- and inter-assay coefficients of variation were, respectively, 10.5–11.1% and 9.4–12.8%, and the lower limit of quantification was 0.2 pmol/l. Testosterone was measured by LC-MS/MS, with the lower limit of detection of 0.08 nmol/l; the inter-assay CV was 8.43% at 0.06 nmol/l, 2.64% at 5.17 nmol/l, and 2.64% at 28.04 nmol/l, and the intra-assay CV was 2.09%, 3.67%, and 1.64% at the mentioned concentrations, respectively.

### Definitions and outcome measures

#### Hypogonadotropic hypogonadism (HH) classification

Subjects were classified as having congenital HH (CHH) if they had a supporting medical history and/or a confirmed deleterious genetic variant, along with low LH, FSH, testosterone, AMH, and/or inhibin B serum concentrations compared to sex and age-matched reference values. Those with an associated abnormal sense of smell, absence of olfactory bulbs on MRI or loss-of-function variants in the *ANOS1* gene were diagnosed with Kallmann syndrome (KS). Patients with a personal history of a clear disruptive condition affecting the hypothalamic pituitary-gonadal axis, such as central nervous system tumour development and/or intracranial surgery, alongside low LH, FSH, and testosterone serum concentrations compared to sex and age-matched reference values, were classified as acquired HH (AHH). Patients with absence of spontaneous testicular development (TV <4 ml) prior to gonadotropin therapy were diagnosed with complete HH (cHH), while those with HH who were able to initiate puberty spontaneously (TV ≥4 ml), but subsequently experienced arrested puberty, were diagnosed with partial HH (pHH).

#### Clinical features

Cryptorchidism was defined as the absence of at least one testis from the scrotum or a personal history of orchidopexy registered in the medical records. Micropenis was defined based on stretched penile length >2 standard deviations below the mean for age (Stancampiano *et al.*, 2022) and microorchidism was at least one testis with volume ≤1 ml before therapy with recombinant gonadotropins. Testicular volume was measured by comparison with Prader’s orchidometer and the larger of the two testes was reported (maximum testis volume). Height was measured using a wall-mounted stadiometer, and weight was measured using an electronic calibrated scale. The presence of abnormal sense of smell was determined based on self-reported perception.

### Treatment schedules for HH

Gonadotropin treatment for pubertal induction for HH started at the RLH in 2010 and has since been added to both Barts Health and UCLH Trust formularies. Local consensus guidelines were developed based on existing literature on treatment with gonadotropins, with the protocol available on the British Society of Paediatric Endocrinology and Diabetes (BSPED) website (Dunkel *et al.*, 2021). Patients with cHH are commenced on pre-treatment with rFSH, followed by treatment with combined rFSH and hCG. rFSH is initiated at a dose of 75–150 IU subcutaneously (SC) 2–3 times a week for 2 months. Close monitoring of serum FSH is done to ensure target levels of 4–6 IU/l are achieved and dose adjustments are made as needed, with doses up to 225 IU rFSH 2–3 times per week. While on rFSH monotherapy, patients may also receive intramuscular or topical testosterone, until the point of starting hCG therapy (with subsequent endogenous testosterone production), when exogenous testosterone is discontinued. Following 2–3 months of serum FSH concentration established in range (4–6 IU/l), hCG 500–2000 IU SC once-twice weekly is added, with the dose titrated based on peak testosterone concentrations, aiming for 10 nmol/l. Patients with pHH are commenced on hCG monotherapy at 500–2000 IU SC once or twice weekly, with the dose titrated as for cHH. If spermatogenesis, or testes volumes of 10–12 ml, is not achieved by 6–12 months on hCG monotherapy, rFSH 150–225 IU SC 2–3 times per week is added. Duration of therapy is typically 9–18 months for pHH and 12–24 months for individuals with cHH, although a longer duration may be required in younger patients or those with severe microorchidism or cryptorchidism. Completion of therapy with gonadotropins is defined by the patient converting from gonadotropin to exogenous testosterone therapy. For further details of the protocol, see Supplementary File S1.

### Outcomes

Primary outcomes of the treatment regimen were semen characteristics (semen volume, and sperm count, motility, progression and morphology) and attainment of maximal TV. Secondary outcomes were changes in serum concentration of FSH, testosterone, AMH and inhibin B after recombinant gonadotropin therapy, duration and doses of rFSH and hCG at the time of each main outcome, and correlation between inhibin B and maximum sperm count. Treatment tolerability and adverse events were assessed by direct questioning and clinical examination for acne, gynaecomastia and injection reactions, as well as an open question on other adverse effects noted by the patient and/or carer, during routine visits to the endocrinologist. Adherence was monitored by measurement of serum concentrations of FSH and testosterone (the latter for adherence to hCG therapy), direct questioning, and frequency of prescriptions.

### Statistical analysis

All statistical analyses were performed using GraphPad Prism version 10.2.1 for Windows (GraphPad Software, San Diego, CA, USA). Data distribution was assessed for normality using the Shapiro-Wilks test. Any missing data were excluded. For values below the assay limit of detection, these were approximated at half the limit of detection for the purposes of calculations of averages and statistical analyses (Whitcomb and Schisterman, 2008; Aung *et al.*, 2023). Continuous variables were summarized as mean (SD) or median (range) according to normality and categorical data as count (percentage). For assessing changes before and after recombinant gonadotropin therapy, we used the Wilcoxon matched pairs signed rank test with its 95% confidence intervals (CI). For the analysis of correlations, we performed a Spearman test. For all the comparisons, the level of significance was set at *P* < 0.05.

## Results

### Participants

**Figure 1.**
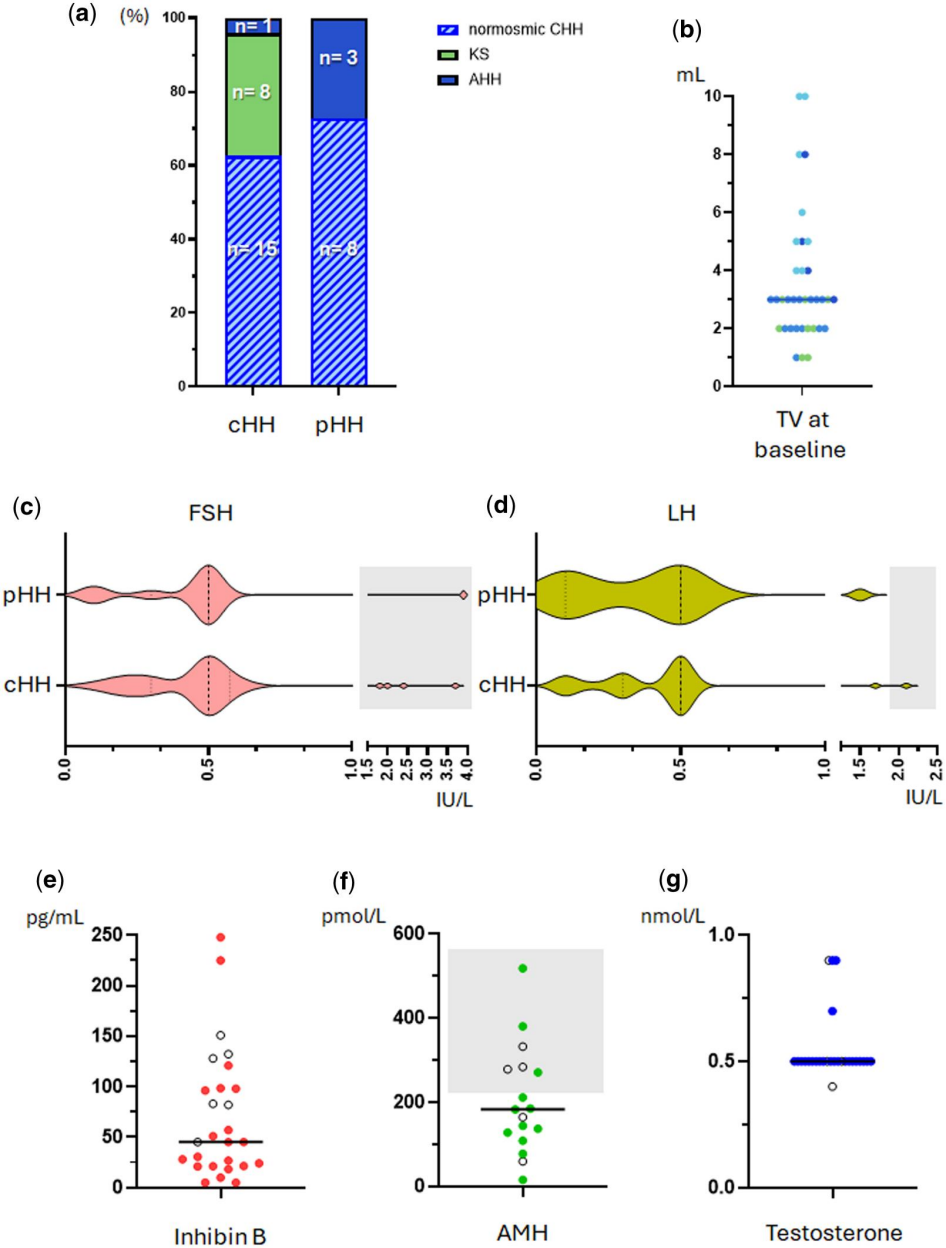
**Patient characteristics prior to gonadotropin therapy.** (**a**) Sub-category of diagnosis; (**b**) distribution of testicular volume measured by Prader orchidometer, colours match the diagnostic categories in (a) (blue—cHH, green—KS, sky blue—pHH, dark blue—AHH); (**c–g**) serum concentrations of (c) FSH (IU/l), (d) LH (IU/l), (e) inhibin B (pg/ml), (f) AMH (pmol/l), and (g) testosterone (nmol/l). Violin plots: dashed lines represent median values. Dotted lines represent the first and third quartiles. Scatter dot plots: open circles indicate patients with pHH; closed circles indicate those with cHH. Horizontal bars represent median values. Shadings represent sex and age-matched normal values for FSH and LH, and age and Tanner stage-matched normal values for AMH. No shading is shown for testosterone or inhibin B as patients' values all were below the lower limit of reference range (Supplementary Table S4) (Bergadá *et al.*, 1999; Grinspon *et al.*, 2011; 2012). AHH, acquired hypogonadotropic hypogonadism; KS, Kallmann syndrome; cHH, complete hypogonadotropic hypogonadism; pHH, partial hypogonadotropic hypogonadism; CHH, congenital hypogonadotropic hypogonadism; TV, testicular volume; FSH, follicle-stimulating hormone; LH, luteinizing hormone; AMH, anti-Müllerian hormone.

There were 36 patients eligible for this treatment regimen. After data evaluation, one subject was excluded due to less than two months adherence to gonadotropin therapy. Of the 35 male patients with HH who received recombinant gonadotropin therapy, 23 had normosmic CHH (65.7%), further subdivided into those with partial (pHH n = 8; 22.9%) and those with complete gonadotropin deficiency (cHH n = 15; 42.9%). An additional eight patients (22.9%) had Kallmann syndrome (cHH with anosmia). The remaining four patients (11.3%) had non-congenital forms of HH (AHH) developed after intracranial surgery for Cushing disease or craniopharyngioma (Fig. 1a). Three of these patients with AHH had pHH at baseline assessment and one had cHH. Median TV before recombinant gonadotropin therapy was 3 ml (range: 1–10) (Fig. 1b).

Baseline clinical characteristics of the cohort of patients included are shown in Table 1 and Supplementary Table S1. Main anthropometric characteristics before recombinant gonadotropin therapy were similar between subjects with cHH and pHH. However, the proportion of subjects with cryptorchidism or micropenis was greater in the KS group than in the normosmic CHH group. Additionally, baseline maximal TV and inhibin B serum concentration were lower in patients with KS than in those with normosmic CHH. Delayed puberty (n = 18/35; 51.4%), alone or in combination with any of the red flag signs (cryptorchidism, micropenis, and/or microorchidism) (Persani *et al.*, 2021), was the most common reason for referral to a paediatric endocrinologist, followed by the isolated presence of red flag signs (n = 6/35; 17.1%). Cryptorchidism (n = 12/35; 34.2%) alone or in combination with micropenis, was the most prevalent red flag sign, even when assessing cHH (n = 10/24; 41.6%) and pHH (n = 2/11; 18.0%), independently. More than 50% (n = 18/35) of the cohort exhibited an exclusively hypogonadic phenotype (non-syndromic HH), whereas intellectual disability or neurobehavioural disorders (n = 4/35; 11.4%) were the most common features among those with a broader phenotype. Only eight patients (22.9%) had Kallmann syndrome; all of them had cHH and self-reported anosmia or hyposmia. Additionally, two had abnormal olfactory findings on MRI, and two others had deletions affecting the *ANOS1* gene. A positive family history of delayed puberty or HH in first-degree relatives was reported in nearly 50% (n = 15/31) of the patients with CHH, with maternal delayed puberty being most prevalent.

**Table 1.** Red flag signs, testicular volume, and median serum hormone concentrations in Kallmann syndrome, normosmic CHH, and AHH at baseline.

	n	KS	n	Normosmic CHH	*P*	n	AHH
Cryptorchidism, n (%)	8	6 (75.0)	23	4 (17.4)	0.02	4	0 (0.0)
Micropenis, n (%)	8	5 (62.5)	23	6 (26.1)	0.03	4	0 (0.0)
Microorchidism, n (%)	8	0 (0.0)	23	1 (4.3)	N.A.	4	0 (0.0)
Maximal TV (ml)^#^	8	2.0 (1.0–3.0)	23	3.0 (1.0–10.0)	0.03	4	4.5 (3.0–8.0)
Serum FSH (IU/l)^#^	8	0.4 (0.2–1.8)	23	0.5 (0.1–3.7)	0.25	4	0.5 (0.3–3.9)
Serum LH (IU/l)^#^	8	0.4 (0.1–0.5)	23	0.5 (0.1–2.1)	0.51	4	0.5 (0.3–1.5)
Serum inhibin B (pg/ml)^#^	6	22.5 (4.9–30.6)	18	53.9 (9.8–225.2)	0.01	3	98.4 (98.0–248.0)
Serum AMH (pmol/l)^#^	3	136.7 (77.4–270.6)	13	182.7 (15.4–518.0)	0.52	1	211.0 (211.0)
Serum testosterone (nmol/l)^#^	6	0.5 (0.5–0.7)	17	0.5 (0.4–0.9)	1.00	4	0.5 (0.5)

# Data are presented as median and range.

*P* values represent the statistical significance after performing a Mann–Whitney test (continuous variables) or a Fisher’s exact test (dichotomous variables) between the group of patients with KS and those with normosmic CHH.

KS, Kallmann syndrome; normosmic CHH, normosmic congenital hypogonadotropic hypogonadism; AHH, acquired hypogonadotropic hypogonadism; TV, testicular volume; FSH, follicle stimulating hormone; LH, luteinizing hormone; AMH, anti-Müllerian hormone; N.A, not assessed.

There were 22 (62.9%) patients who received pre-treatment with exogenous testosterone, whilst the remaining 13 (37.1%) were treatment naïve before the initiation of gonadotropin therapy.

### Reproductive axis hormone levels

Prior to exogenous hormone therapy, median serum concentrations of FSH (0.5 IU/l [range: 0.1–3.9]), LH (0.5 IU/l [range: 0.1–2.1]), testosterone (0.5 nmol/l [range: 0.4–0.9]) and inhibin B (45.0 pg/ml [range: 4.9–248.0]) were low compared to the sex and age matched healthy population, as was AMH (182.7 pmol/l [range: 15.4–518.0]) compared to sex and Tanner-stage matched healthy references (Bergadá *et al.*, 1999; Grinspon *et al.*, 2011, 2012) (Fig. 1c–g). In addition, serum concentrations of FSH, LH, AMH, and testosterone were similar between patients with severe HH (cHH + KS) and those with a milder phenotype (pHH + AHH). However, inhibin B serum concentration was lower in the former group compared to those with less severe forms of HH (median inhibin B serum concentration: 27.35 pg/ml [range: 4.9–225.2] and 98.4 pg/ml [range: 45–248.0] respectively; *P* = 0.0007).

### Recombinant gonadotropin therapy

Of the 35 patients treated with exogenous gonadotropins, 34 received a combination of rFSH and hCG. Of these, 22 subjects (62.9%) initially started with rFSH monotherapy as pre-treatment with hCG added later, whereas 12 patients (34.3%) did not qualify for rFSH pre-treatment due to pHH, according to our protocol (Supplementary File S1). The only patient who received hCG monotherapy, who did not require additional rFSH later in his treatment course, exhibited arrested puberty (baseline TV right 6 ml, left 8 ml) after surgery for craniopharyngioma in early adolescence, without major compromise of the FSH–Sertoli cell axis (serum concentrations of FSH 3.9 IU/l and inhibin B 248 pg/ml prior to starting therapy).

**Figure 2.**
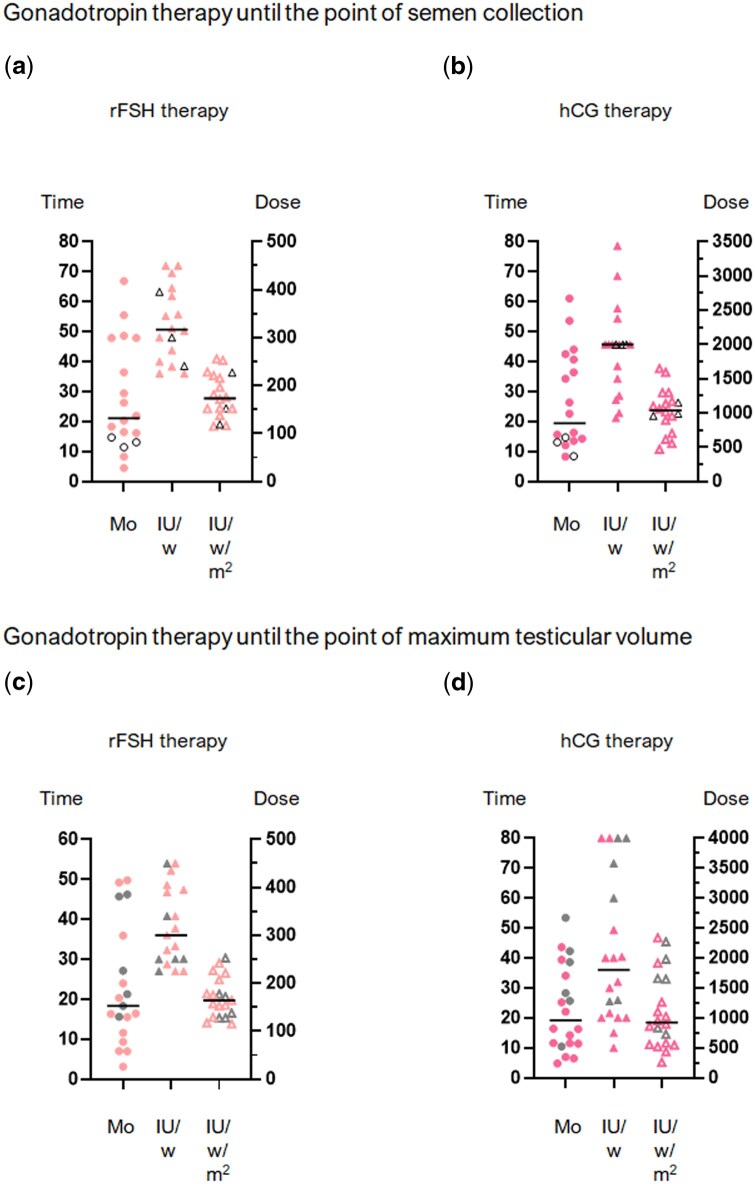
**Treatment regimens at the time of semen analysis and maximum testicular volume.** Duration and dose of recombinant FSH and hCG up until the point of semen sample collection (**a**, **b**) or maximum testicular volume (**c**, **d**). Pale pink symbols represent data from recombinant FSH therapy (a, c), while dark pink symbols represent data from hCG therapy (b, d). Circles indicate treatment duration (months), closed triangles represent doses in IU/week, and open triangles represent doses in IU/week/m^2^. Open black symbols indicate the therapeutic regimen in patients with azoospermia (a, b), while grey symbols represent the therapy regimen in patients whose maximum testicular volume was below the cohort median (c, d). Horizontal bars represent median values. Mo, months; IU/w, International Units/week; IU/w/m^2^, International Units/week/squared metres; rFSH, recombinant follicle stimulating hormone; hCG, human chorionic gonadotropin.

Of 35 patients treated with gonadotropins, 19 (54.3%) had completed therapy and achieved their maximal TV at the time of data analysis, and 18/19 (94.7%) provided a semen sample for analysis. The median duration and dose of rFSH over the treatment course until the time of semen sample (n = 18) were 21.1 months (range: 4.5–66.9) and 316.8 IU/week (range: 225–450), respectively. Additionally, the median duration and dose of hCG until the time of semen sample was 19.5 months (range: 8.3–61.1) and 2000 IU/week (range: 927.4–3442), respectively (Fig. 2a and b). For those patients who had completed gonadotropin therapy (n = 19), the median durations and doses received up until the time of the achievement of maximal TV was 18.3 months (range: 3.1–49.8) and 300 IU/week (range: 225–450) for rFSH and 19.3 months (range: 4.8–53.4) and 1801 IU/week (range: 500–4000) for hCG (Fig. 2c and d). The treatment duration and doses of rFSH and hCG received by patients with CHH or the full cohort (including those with acquired disease) were similar (Supplementary Table S2), whereas the rFSH and hCG treatment duration was longer in patients with KS than in those with normosmic CHH (Supplementary Table S3).

### Clinical and biochemical changes following recombinant gonadotropin therapy

**Figure 3.**
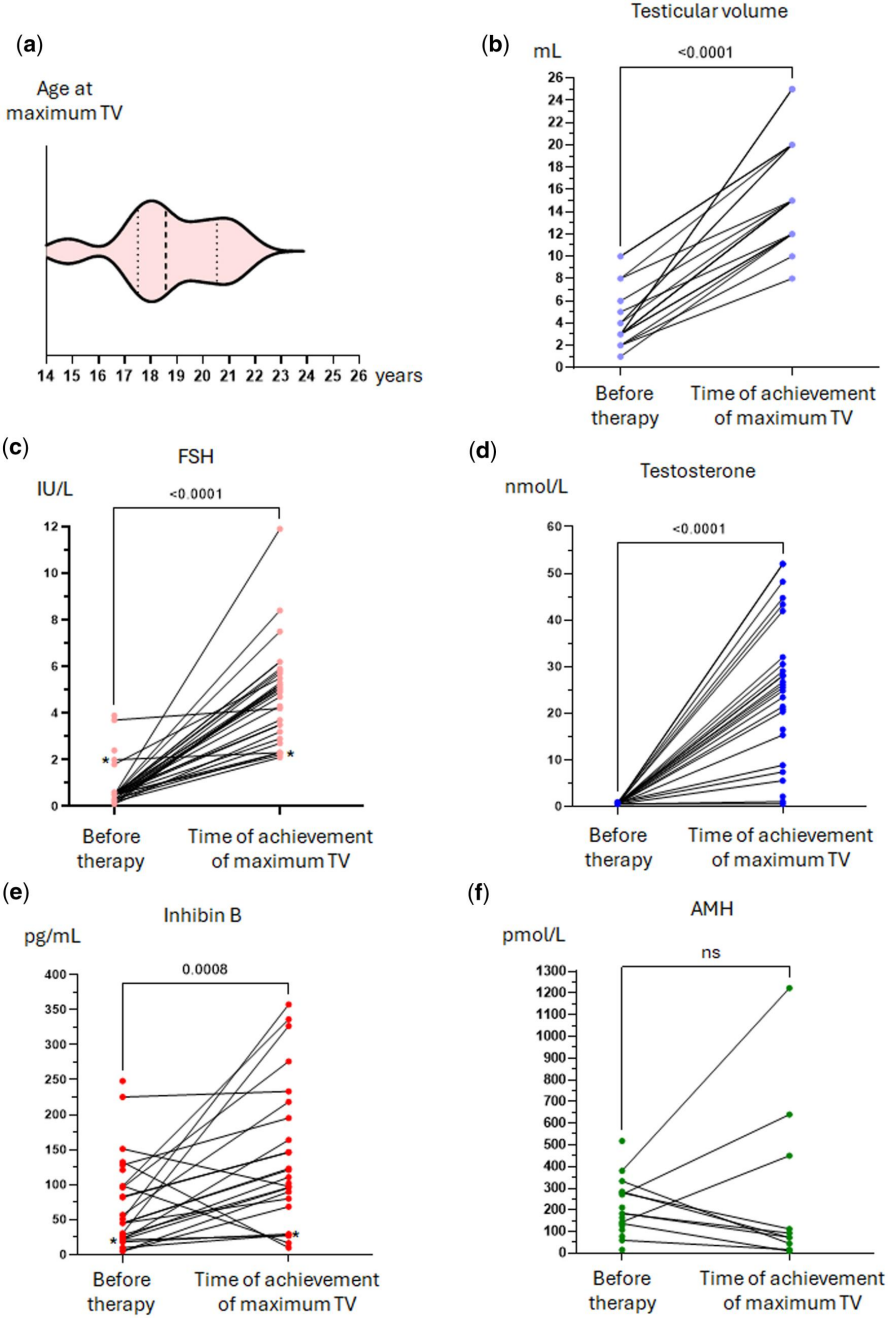
**Pre- and post-gonadotropin therapy analysis of clinical and biochemical characteristics.** (**a**) Age distribution at the time of achievement of maximum testicular volume; (**b**) testicular volume distribution before and after gonadotropin therapy; (**c**–**f**) serum concentrations of (c) FSH (n = 31), (d) testosterone (n = 24), (e) inhibin B (n = 24), and (f) AMH (n = 10) before and after gonadotropin therapy. Pre-/post-statistical analysis was performed using Wilcoxon matched pairs signed rank test. Asterisks (*) represent patients with low adherence to therapy. Dashed line represents median values. Dotted line represents the first and third quartiles. TV, testicular volume; FSH, follicle stimulating hormone; AMH, anti-Müllerian hormone.

The median age at the time of achievement of maximal TV was 18.6 years (range: 14.5–21.9) (Fig. 3a). There was a significant increase in TV after recombinant gonadotropin therapy compared to baseline (median basal TV: 3 ml [range: 1.0–10.0]), with a median change of 10.5 ml after therapy (95% CI 9.5–13.6; *P* < 0.0001) (Fig. 3b). The median maximal TV after the completion of gonadotropin therapy was 15 ml (range: 8–25). No differences were observed in any of the above outcomes when comparing patients with CHH with the full cohort of patients (Supplementary Table S2), whereas the maximal TV achieved in males with normosmic CHH was greater than in those with KS (Supplementary Table S3), despite both groups showing a similar increase in testicular volume with treatment. Six patients achieved a TV below 15 ml: one had 8 ml testes post-therapy, another patient had 10 ml, and the remaining four subjects achieved TV of 12 ml. Of the 19 patients who completed therapy with gonadotropins, 18 (94.7%) reached a Tanner genital stage of ≥3 (Tanner stage 3: n = 1; 5.2%, Tanner stage 4: n = 5; 26.4%, and Tanner stage 5: n = 12; 63.2%). The only patient with Tanner genital stage 2 had poor adherence to therapy. FSH, testosterone and inhibin B serum concentrations significantly increased after recombinant gonadotropin therapy, whereas AMH was more variable (FSH median increase: 4.4 IU/l; 95% CI 3.4–5.0; *P* < 0.0001; Testosterone median increase: 25.7 nmol/l; 95% CI 19.8–31.5; *P* < 0.0001; Inhibin B median increase: 67.7 pg/ml; 95% CI 18.4–86.7; *P* = 0.0008; AMH median change: −101.0 pmol/l; 95% CI −211.7–369.2; *P* = 0.9999) (Fig. 3c–f). For three patients, inhibin B decreased after treatment as compared to baseline, the reason for which was not clear but may reflect a physiological decrease at the end of puberty (from Tanner G3 to Tanner G4-5) seen in some men (Borelli-Kjær *et al.*, 2024), or that only two measurements have not captured the trajectory of the inhibin B concentrations during the treatment (which may have increased and then fallen later in the treatment course). The variability in AMH change with treatment is likely to reflect variation in maturation of the Sertoli cell population between cohort individuals, as AMH rises with Sertoli cell stimulation by rFSH, but then falls with Sertoli cell differentiation driven by intratesticular testosterone later in puberty (Grinspon *et al.*, 2011). The median increase in inhibin B serum concentration after therapy for the subset of six patients who achieved a TV below 15 ml was 65.9 pg/ml (range: 35.0–84.7). This was comparable to the inhibin B increase in those with a TV ≥15 ml (70.6 pg/ml; *P* = 0.911). The assessment of serum hormone concentrations before and after excluding patients with AHH showed no differences between both groups (Supplementary Table S2), nor were there any differences when comparing the KS group with the normosmic CHH group (Supplementary Table S3).

### Semen assessment

**Figure 4.**
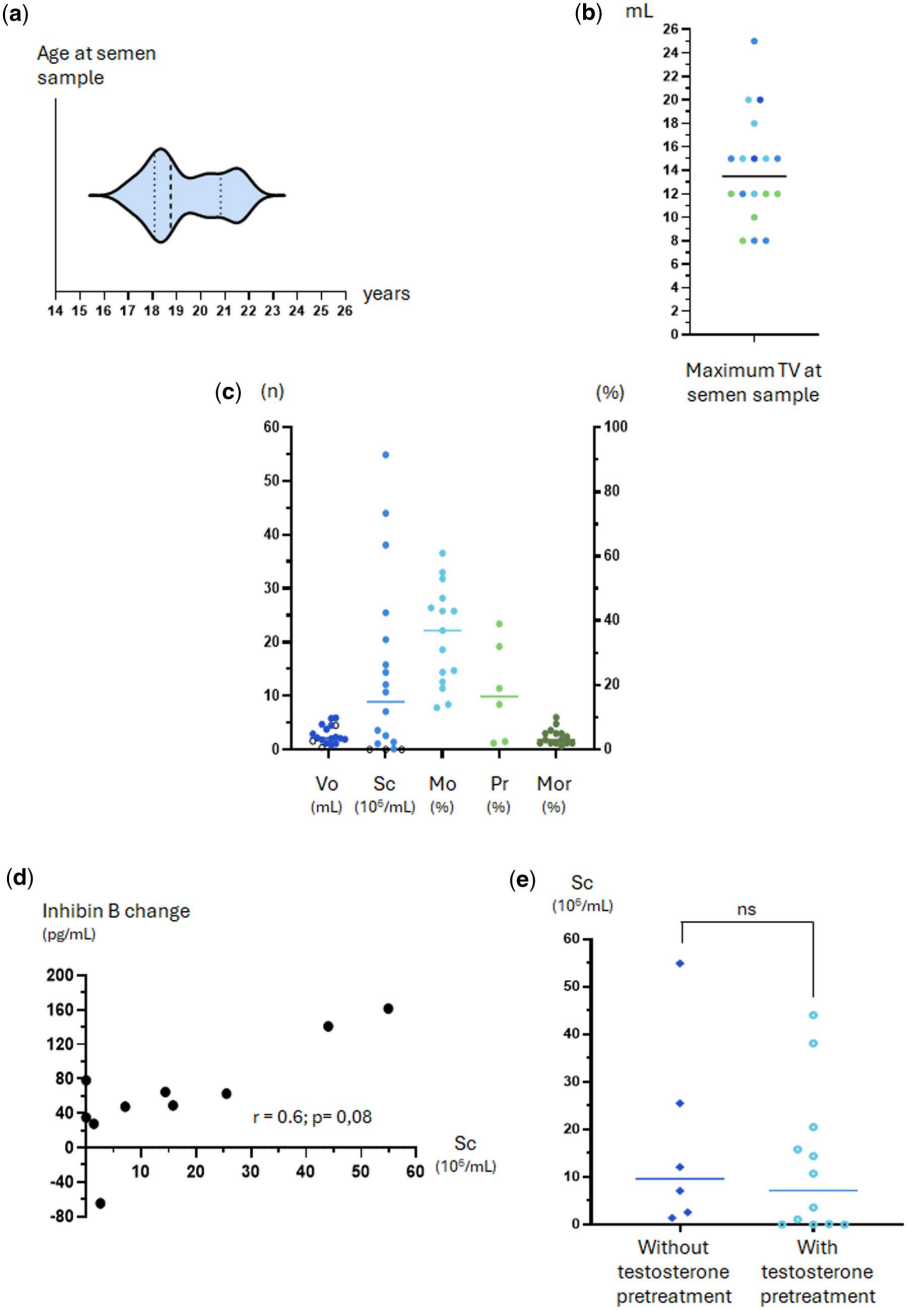
**Semen sample analysis.** (**a**) Age distribution at the time of semen sample collection; (**b**) testicular volume distribution at the time of semen sample collection, colours match the diagnostic categories in (a); (**c**) assessment of semen quality; open symbols indicate patients with azoospermia. TV—mL, testicular volume; Vo—mL, volume; Sc—10^6^/ml, sperm count; Mo—%, motility; Pr—%, progression; Mor—%, normal morphology. (**d**) correlation between maximum sperm count and increase in inhibin B serum concentrations after therapy; the analysis of correlation was performed using the Spearman test; (**e**) sperm count at semen analysis for patients with and without testosterone pre-treatment. Median values were compared using the Mann–Whitney test.

Of the 19 eligible patients, 18 (94.7%) provided a semen sample for analysis, with one patient reporting being emotionally unprepared for semen collection. The median age and TV at the time of semen sample was 18.8 years (range: 17.0–21.9) and 13.5 ml (range: 8.0–25.0), respectively (Fig. 4a and b). The median values for semen volume, sperm count, motility, progression and normal morphology were 2.1 ml (range: 0.4–5.9), 8.9 × 10^6^/ml (range: 0.0–54.9), 37.0% (range: 13.0–61.0), 16.5% (range: 2.0–39.0) and 3.0% (range: 1.0–10.0), respectively (Fig. 4c), and showed no differences after excluding patients with AHH (Supplementary Table S2), comparing KS with normosmic CHH patients (Supplementary Table S3) or pHH with cHH (data not shown). According to 2021 WHO criteria (Baldi *et al.*, 2022), more than one-third of the patients had semen quality comparable to that of healthy adult men. Assessed parameters were above the WHO lower limit for healthy men for semen volume in 77.8% (14/18), sperm count 37.5% (6/16), motility 46.7% (7/15), progression 33.3% (2/6), and normal morphology 46.7% (7/15) of patients. Three patients had azoospermia on semen analysis after rFSH therapy, with treatment duration from 11 to 14 months and doses from 240 to 395 IU/week. The average hCG dose for these three patients was 2000 IU/week, with hCG therapy duration from 8 to 15 months. Two of the three patients with azoospermia received pre-treatment with rFSH, while the remaining patient received combined therapy with rFSH + hCG. Gonadal axis impairment among patients with azoospermia was variable, as were serum concentrations of FSH, LH, testosterone, AMH, and inhibin B when compared to the cohort median values. The three patients with azoospermia had TV <4 ml at the time of treatment initiation and two of these patients exhibited poor adherence to therapy and had only achieved TV of 8 ml at the time of semen sample collection. The remaining patient with azoospermia had a TV of 12 ml at the time of semen collection, below the cohort median of 15 ml. The change in inhibin B serum concentration pre-/post-therapy showed a trend towards a positive correlation with sperm count (r = 0.6; *P* = 0.08) (Fig. 4d). No difference was observed after comparing median sperm count in patients without (6/18) and with (12/18) testosterone pre-treatment (without testosterone pre-treatment: 9.6 × 10^6^; range: 1.4–54.9; with testosterone pre-treatment: 7.1 × 10^6^; 0.0–44.0. *P* = 0.42) (Fig. 4e).

### Safety and tolerability of gonadotropin treatment

Tolerability of treatment and potential occurrence of adverse events were monitored at each clinic visit through open questions during routine visits to the endocrinologist. All patients had normal haemoglobin, renal function, lipids, and hepatic function throughout treatment with gonadotropins. Treatment was well-tolerated with just one patient opting to stop hCG therapy due to cystitis. The majority of patients (32 of 35) reported self-injecting, while three patients had injections administered by a parent. There were no adverse effects related to acne, gynaecomastia or injection site issues reported.

## Discussion

Increasing numbers of centres internationally are offering gonadotropin treatment to adolescent and young adult men for full pubertal induction, instead of using exogenous testosterone to develop secondary sexual characteristics (Howard and Dunkel, 2016). These centres are driven by the goal of improving outcomes for patients with HH, particularly with respect to achieving timely and effective replacement in puberty. Evidence supports the use of central gonadotropin replacement of puberty, in particular through administration of rFSH alongside hCG or LH, to improve not only secondary sexual characteristics and psychological well-being (Shiraishi *et al.*, 2014), but also testicular maturation and spermatogenesis (Rohayem *et al.*, 2017; Dwyer *et al.*, 2019; Alexander *et al.*, 2024). However, no standardized protocols exist, and the choice of gonadotropin, doses, timing, and availability of treatment varies widely.

Hence, we set out to assess the outcomes from the use of our local consensus protocol (Supplementary File S1) in two tertiary UK centres. In this retrospective observational review of male adolescents with a spectrum of congenital and acquired HH and varying degrees of gonadotropins deficiency (partial versus complete), we describe that the treatment with rFSH and hCG is safe and effective for inducing puberty to its final stages (Tanner 4–5), and that findings from adult males are also applicable to adolescent and young adult patients. Other studies have focused on spermatogenesis and TV changes following the use of recombinant or urinary gonadotropin therapy. However, many of these studies included adult patients seeking fertility rather than young males referred for lack of pubertal development (Nachtigall *et al.*, 1997; Boepple *et al.*, 2008; Farhat *et al.*, 2010; Hosseinifar *et al.*, 2013; Sanyal and Chatterjee, 2016; Nie *et al.*, 2021), included other aetiologies besides HH (Rohayem *et al.*, 2017), and/or used different treatment regimens such as GnRH pulsatile therapy (Aydogdu *et al.*, 2013; Gu *et al.*, 2021; Nie *et al.*, 2021; Shankar *et al.*, 2022). Rastrelli *et al.* assessed the event rate of producing at least one spermatozoa and the sperm count following gonadotropin therapy, as reported in 49 studies. The event rate achieved was significantly lower in individuals with pre-pubertal onset of HH compared to the mixed population (pre-pubertal and post-pubertal) (0.68 vs. 0.84; *P* = 0.011), and in those who received hCG monotherapy compared to those treated with rFSH + hCG (0.47 vs. 0.80; *P* < 0.0001). Sperm count was also significantly lower in the pre-pubertal subgroup of patients (Rastrelli *et al.*, 2014). Although this meta-analysis summarizes the medical experience with gonadotropin therapy up until 2014, it included studies with different treatment regimens, mostly focusing on adult males seeking fertility rather than pubertal induction. We report successful spermatogenesis, with detectable sperm after therapy with gonadotropins, in over 80% of the patients studied (15/18). Our results are consistent with the highest rates of spermatogenesis reported in the literature using combinations of rFSH and hCG (Zacharin *et al.*, 2012; Cangiano *et al.*, 2021; Alexander *et al.*, 2024) and appear to show superiority compared to previous experiences using hCG monotherapy (Alexander *et al.*, 2024). While our protocol (Supplementary File S1) suggests a starting regime of hCG monotherapy in patients with pHH, addition of rFSH is used if adequate testicular volumes (of 10 ml or more) or spermatogenesis have not been achieved on hCG monotherapy after 6–12 months. In fact, only one patient received hCG monotherapy and all other patients with pHH received additional rFSH. Accordingly, on the basis of this study, we have made updates to our protocol and this updated version is included in Supplementary File S1.

Our experience is highly suggestive that rFSH is also required to complete puberty in males with pHH, but evidence from prospective randomized trials is needed to confirm this. Evidence specifically for the benefit of pre-treatment with rFSH during pubertal induction for patients with complete or severe disease is not yet clear. In alignment with previous reports (Dwyer *et al.*, 2024), our data demonstrate that patients with cryptorchidism, absent spontaneous puberty, low baseline inhibin B, and pathogenic *ANOS1* variants such as in the KS subgroup of our cohort, require a longer duration of combined gonadotropins and do not achieve as high TV as those with less severe disease. However, their rate of spermatogenesis and median sperm count were comparable to the normosmic CHH group, despite with a lower average sperm count. Recently, the first long-term follow-up to fertility for patients treated with gonadotropins for pubertal induction has shown that, despite a high variability in TV, inhibin B concentrations and required length of gonadotropin therapy to achieve fertility, the fertility outcomes in these individuals were very encouraging (Grob *et al.*, 2024). Overall, for the majority of patients with HH, pubertal induction with gonadotropins is highly successful in achieving spermatogenesis.

Only three patients in our cohort had no detectable sperm count in their ejaculate. These three patients received a shorter course of therapy (therapy duration for rFSH ranged from 11 to 14 months and for hCG ranged from 8 to 15 months, although with a similar dose of rFSH and hCG (rFSH doses ranged from 240 to 395 IU/week, while the hCG dose was 2000 IU/week) compared to the cohort median values, probably highlighting the necessity of a minimum treatment duration threshold for effective induction of spermatogenesis. Additionally, our study demonstrates a greater increase in TV after combined therapy with rFSH and hCG than that previously reported with hCG monotherapy (Yang *et al.*, 2012; Aydogdu *et al.*, 2013; Bayram *et al.*, 2016). Notably, our cohort exhibited a greater increase in TV compared to previous reports using combinations of rFSH and hCG, possibly reflecting more heterogeneity in the therapeutic regimens used by other groups (Burgués and Calderón, 1997; Barrio *et al.*, 1999; Bouloux *et al.*, 2002). Patients in our cohort reported good levels of satisfaction with their achieved TV, although these data were not formally collected.

We describe a trend to positive correlation between the increase in inhibin B serum concentrations after therapy and maximum sperm count. This finding supports the idea that pre-treatment with rFSH in those with small testes volumes emulates endogenous FSH action during both mini-puberty and early pubertal stages, promoting Sertoli cell proliferation before their terminal differentiation, which is crucial for germ cell proliferation and differentiation during spermatogenesis (Cannarella *et al.*, 2024).

It is well established that male adolescents and young adults with delayed puberty experience significant psychological distress, low self-esteem, social isolation, fewer sexual relationships, and lower quality of life (Dwyer *et al.*, 2019, 2021). We observed similar sperm counts between those who received exogenous testosterone treatment prior to gonadotropin therapy and those who began directly on gonadotropins for pubertal induction. This finding is aligned with previous data (Rastrelli *et al.*, 2014) and may help to reassure paediatric endocrinologists with limited access to rFSH or hCG that the use of exogenous testosterone to induce androgen-dependent changes in patients seeking treatment for pubertal delay is unlikely to compromise spermatogenic potential, should gonadotropins become available at a later stage (Rohayem *et al.*, 2017).

The population of patients with HH described in our study represents its primary strength. First, we included patients with diverse forms of HH, thereby providing encouraging results across a variety of subjects with impaired puberty who face increased odds of fertility problems in adulthood. Additionally, our cohort is considerably younger than those in most previous studies. This aspect is of particular interest for several reasons: first, many of the patients in our cohort were able to avoid experiencing pubertal delay and its recognized negative impact on emotional and social well-being. Second, the use of recombinant gonadotropins facilitated Sertoli cell proliferation and facilitated Leydig–Sertoli cell interactions required for semen production, potentially increasing the chances of better fertility results following shorter courses of recombinant gonadotropins in adulthood. Third, our results support that using recombinant gonadotropins for an average of 18–24 months in adolescent patients is safe and well tolerated.

Our work has some limitations. Although we report results based on more standardized treatment schemes than many previous studies in the literature describing paediatric populations, the lack of a prospective design accounts for the dose and duration differences observed in our cohort. Another limitation is the relatively low representation of patients with AHH in our study, emphasizing the need to extrapolate these findings with caution in this specific subgroup of adolescent males with HH. The study is also an observational one, therefore meaning that some outcomes (such as inhibin B) were not collected routinely and not reported for all patients. We did not include a comparative cohort treated with testosterone, and many patients also received testosterone prior to their treatment with gonadotropins; although current evidence suggests testosterone treatment does not affect the therapeutic response to gonadotropins (Kumar *et al.*, 2006). Finally, although we reported all long-term data that were available, the long-term effects of early intervention in patients with HH require further evaluation. This is particularly relevant for fertility, where spermatogenesis alone is a substitute marker for the achievement of paternity.

In conclusion, the use of rFSH in combination with hCG is a feasible, well-tolerated, and effective treatment for inducing puberty and promoting spermatogenesis in male adolescents with HH. The use of pubertal induction with these medications reduces the duration and likely increases the effectiveness of future fertility induction protocols in adulthood, positively impacting on quality of life for individuals. Clinical trials, with prospective data collection and ideally direct comparison of protocols using hCG monotherapy, combined therapy, or rFSH pre-treatment, are needed to confirm these results and provide new insight into optimal treatment choices and fertility potential in patients with HH.

## Supplementary Material

hoaf026_Supplementary_Data

## Data Availability

The data underlying this article will be shared on reasonable request to the corresponding author.
