# Supplementary material for: Effect of pubertal induction with combined gonadotropin therapy on testes development and spermatogenesis in males with gonadotropin deficiency: a cohort study
Source: Hum Reprod Open. 2025 May 13;2025(2):hoaf026. doi: 10.1093/hropen/hoaf026 (PMC12132099; doi:10.1093/hropen/hoaf026)
Supplement: hoaf026_Supplementary_Data [file hoaf026_supplementary_data.zip › HRO-25-0015-R2-SuppTables1-4.docx]

**Supplementary Tables**

Supplementary Table S1. Baseline characteristics of the study cohort patients

|  | n | **Full cohort** | n | **cHH cohort** | n | **pHH cohort** |
| --- | --- | --- | --- | --- | --- | --- |
| Age (yr)^#^ | 35 | 15.8 (11.8 – 22.7) | 24 | 15.6 (11.8 – 22.7) | 11 | 16.2 (13.4 – 19.5) |
| Body weight (Kg)^#^ | 35 | 58.9 (36.3 – 119.5) | 24 | 65.8 (36.3 – 119.5) | 11 | 58.2 (36.6 – 87.5) |
| Body weight (SD)^#^ | 35 | 0.4 (-3.5 – 4.1) | 24 | 0.4 (-2.6 – 4.1) | 11 | 0.1 (-3.5 – 2.3) |
| Height (cm)^#^ | 35 | 165.8 (135.8 – 183.5) | 24 | 166.8 (135.8 – 183.5) | 11 | 163.2 (147.4 – 179.7) |
| Height (SD)^#^ | 35 | -0.5 (-2.5 – 1.3) | 24 | -0.3 (-2.5 – 1.3) | 11 | -1.2 (-2.4 – 0.8) |
| BMI (kg/m^2^)^#^ | 35 | 22.4 (15.1 – 40.3) | 24 | 23.5 (16.3 – 40.3) | 11 | 21.6 (15.1 – 33.9) |
| BMI (SD)^#^ | 35 | 0.7 (-3.0 – 3.7) | 24 | 1.0 (-1.8 – 3.7) | 11 | 0.0 (-3.0 – 3.1) |
| Reason for referral, n (%) |  |  |  |  |  |  |
| - DP | 35 | 11 (31.4) | 24 | 9 (37.4) | 11 | 2 (18.2) |
| - Red flags | 35 | 6 (17.1) | 24 | 5 (20.8) | 11 | 1 (9.0) |
| - DP + Red flags | 35 | 7 (20.0) | 24 | 6 (25.0) | 11 | 1 (9.0) |
| - DP + SS | 35 | 4 (11.4) | 24 | 1 (4.2) | 11 | 3 (27.4) |
| - Red flags + SS | 35 | 1 (2.9) | 24 | 1 (4.2) | 11 | 0 (0.0) |
| - DP + red flags + SS | 35 | 1 (2.9) | 24 | 1 (4.2) | 11 | 0 (0.0) |
| - Additional pituitary hormone deficiencies | 35 | 2 (5.7) | 24 | 0 (0.0) | 11 | 2 (18.2) |
| - Arrested puberty | 35 | 2 (5.7) | 24 | 0 (0.0) | 11 | 2 (18.2) |
| - IH signs | 35 | 1 (2.9) | 24 | 1 (4.2) | 11 | 0 (0.0) |
| Red flags, n (%) |  |  |  |  |  |  |
| - Cryptorchidism | 35 | 6 (17.1) | 24 | 5 (20.8) | 11 | 1 (9.0) |
| - Micropenis + cryptorchidism | 35 | 6 (17.1) | 24 | 5 (20.8) | 11 | 1 (9.0) |
| - Micropenis | 35 | 3 (8.6) | 24 | 3 (12.5) | 11 | 0 (0.0) |
| - Microorchidism | 35 | 1 (2.9) | 24 | 1 (4.2) | 11 | 0 (0.0) |
| - None | 35 | 19 (54.3) | 24 | 10 (41.7) | 11 | 9 (82.0) |
| Extragonadal features, n (%) |  |  |  |  |  |  |
| - ID/BD | 35 | 4 (11.4) | 24 | 4 (16.7) | 11 | 0 (0.0) |
| - Synkinesis | 35 | 2 (5.7) | 24 | 2 (8.1) | 11 | 0 (0.0) |
| - Dental abnormality | 35 | 1 (2.9) | 24 | 1 (4.2) | 11 | 0 (0.0) |
| - Renal abnormality | 35 | 1 (2.9) | 24 | 0 (0.0) | 11 | 1 (9.0) |
| - SN hearing loss | 35 | 1 (2.9) | 24 | 1 (4.2) | 11 | 0 (0.0) |
| - Hands anomalies | 35 | 1 (2.9) | 24 | 1 (4.2) | 11 | 0 (0.0) |
| - Ocular anomalies | 35 | 1 (2.9) | 24 | 1 (4.2) | 11 | 0 (0.0) |
| - Other^¥^ | 35 | 6 (17.1) | 24 | 4 (16.7) | 11 | 2 (18.2) |
| - None | 35 | 18 (51.3) | 24 | 10 (41.7) | 11 | 8 (72.8) |
| Anosmia/hyposmia, n (%) |  |  |  |  |  |  |
| - Yes | 35 | 8 (22.9) | 24 | 8 (33.3) | 11 | 0 (0.0) |
| - No | 35 | 27 (77.1) | 24 | 16 (66.7) | 11 | 11 (100.0) |
| Family history, n (%)^Ω^ |  |  |  |  |  |  |
| - DP mother | 31 | 6 (19.4) | 23 | 4 (17.4) | 8 | 2 (25.0) |
| - DP father | 31 | 1 (3.2) | 23 | 1 (4.4) | 8 | 0 (0.0) |
| - DP siblings | 31 | 2 (6.5) | 23 | 1 (4.4) | 8 | 1 (12.5) |
| - HH mother | 31 | 1 (3.2) | 23 | 1 (4.4) | 8 | 0 (0.0) |
| - HH father | 31 | 3 (9.6) | 23 | 2 (8.6) | 8 | 1 (12.5) |
| - HH siblings | 31 | 2 (6.5) | 23 | 2 (8.6) | 0 | (0.0) |
| - Negative family history | 31 | 16 (51.6) | 23 | 12 (52.2) | 4 | (50.0) |

#Data are presented as median and range.

Patients with AHH having a testicular volume < 4 mL before gonadotropin therapy were included in the cHH group, whilst those with a testicular volume ≥ 4 mL were included in the pHH group.

¥Includes asthma (2), eczema (1), epilepsy (1), high-arched palate (1), lupus + diabetes mellitus (1)

ΩPatients with AHH were excluded from the analysis.

cHH: complete hypogonadotropic hypogonadism; pHH: partial hypogonadotropic hypogonadism; yr: years; DP: delayed puberty; SS: short stature; IH: intracranial hypertension; ID/BD: intellectual disability/behavioural disorders; SN: sensorineural; HH: hypogonadotropic hypogonadism.

Supplementary Table S2. Comparison of semen characteristics, testicular volume, and serum hormone concentrations before and after excluding patients with acquired hypogonadotropic hypogonadism (AHH; n=4).

|  | n | **Full cohort** | n | **CHH** | **p** |
| --- | --- | --- | --- | --- | --- |
| Age at semen sample (yr)^#^ | 18 | 18.8 (17.0 – 21.8) | 16 | 19.1 (17.0 – 21.8) | 0.82 |
| TV at semen sample (mL)^#^ | 18 | 13.5 (8.0 – 25.0) | 16 | 12.0 (8.0 – 25.0) | 0.75 |
| Time on rFSH at semen sample (mo)^#^ | 18 | 21.1 (4.5 – 66.9) | 16 | 21.1 (4.5 – 66.9) | 0.93 |
| rFSH average dose at semen sample (IU/w)^#^ | 18 | 316.8 (225.0 – 450.0) | 16 | 329.7 (225.0 – 450.0) | 0.93 |
| Time on hCG at semen sample (mo)^#^ | 18 | 19.5 (8.3 – 61.1) | 16 | 19.5 (8.3 – 61.1) | 0.98 |
| hCG average dose at semen sample (IU/w)^#^ | 18 | 2000.0 (927.4 – 3442.0) | 16 | 2000 (927.4 – 3442.0) | 0.82 |
| Semen volume (mL)^#^ | 18 | 2.1 (0.4 – 5.9) | 16 | 2.1 (0.4 – 5.9) | 1.00 |
| Sperm count (million)^#^ | 18 | 8.9 (0.0 – 54.9) | 16 | 8.9 (0.0 – 54.9) | 0.93 |
| Sperm motility (%)^#^ | 15 | 37.0 (13.0 – 61.0) | 13 | 37.0 (13.0 – 61.0) | 0.97 |
| Sperm progression (%)^#^ | 6 | 16.5 (2.0 – 39.0) | 6 | 16.5 (2.0 – 39.0) | 1.00 |
| Sperm normal morphology (%)^#^ | 15 | 3.0 (1.0 – 10.0) | 13 | 3.0 (1.0 – 10.0) | 0.94 |
| Age at achievement of maximal TV^#^ | 19 | 18.6 (14.5 – 21.9) | 17 | 18.6 (15.2 – 21.9) | 0.70 |
| Increase in TV after therapy (mL)^#^ | 19 | 10.5 (2.0 – 22.0) | 17 | 10.0 (2.0 – 22.0) | 0.65 |
| Maximal TV achieved (mL)^#^ | 19 | 15.0 (8.0 – 25.0) | 17 | 15.0 (8.0 – 25.0) | 0.74 |
| Increase in serum FSH after therapy (IU/L)^Ω^ | 19 | 4.4 (3.4 – 5.0) | 17 | 4.0 (3.2 – 5.0) | 0.80 |
| Increase in serum inhibin B after therapy (pg/mL)^Ω^ | 19 | 67.7 (18.4 – 86.7) | 17 | 67.7 (18.4 – 86.7) | 1.00 |
| Change in serum AMH after therapy (pmol/L) ^Ω^ | 10 | -101.0 (-211.7 – 369.2) | 10 | -101.0 (-211.7 – 369.2) | 0.80 |
| Increase in serum testosterone after therapy (nmol/L) ^Ω^ | 19 | 25.7 (19.8 – 31.5) | 17 | 25.2 (19.8 – 30.0) | 0.80 |

#Data are presented as median and range.

ΩData are presented as median and 95% confidence interval.

P values represent the statistical significance after performing a Mann-Whitney test between the full cohort of patients and the cohort of patients having congenital hypogonadotropic hypogonadism.

CHH: congenital hypogonadotropic hypogonadism; yr: years; mo; months; IU/w: International Units per week; TV: testicular volume; rFSH: recombinant follicle-stimulating hormone; hCG: human chorionic gonadotropin; FSH: Follicle stimulating hormone; AMH: anti-Müllerian hormone.

Supplementary Table S3. Comparison of semen characteristics, testicular volume, and serum hormone concentrations between patients with Kallmann syndrome and those with normosmic congenital hypogonadotropic hypogonadism.

|  | n | **KS** | n | **Normosmic CHH** | **p** |
| --- | --- | --- | --- | --- | --- |
| Age at semen sample (yr)^#^ | 5 | 18.5 (17.9 – 21.6) | 11 | 19.6 (17.0 – 21.8) | 0.74 |
| TV at semen sample (mL)^#^ | 5 | 12.0 (8.0 – 12.0) | 11 | 15.0 (8.0 – 25.0) | 0.08 |
| Time on rFSH at semen sample (mo)^#^ | 5 | 47.9 (18.3 – 66.9) | 11 | 16.5 (4.5 – 36.4) | 0.008 |
| rFSH average dose at semen sample (IU/w)^#^ | 5 | 345.0 (250.4 – 450.0) | 11 | 300.0 (225.0 – 450.0) | 0.60 |
| Time on hCG at semen sample (mo)^#^ | 5 | 44.0 (14.3 – 61.1) | 11 | 15.6 (8.3 – 36.4) | 0.01 |
| hCG average dose at semen sample (IU/w)^#^ | 5 | 2377 (1252 – 3442) | 11 | 2000 (927.4 – 2528) | 0.30 |
| Semen volume (mL)^#^ | 5 | 2.9 (1.1 – 4.5) | 11 | 2.0 (0.4 – 5.9) | 0.53 |
| Sperm count (million)^#^ | 5 | 3.6 (0.1 – 25.5) | 11 | 12.1 (0.0 – 54.9) | 0.64 |
| Sperm motility (%)^#^ | 5 | 43.0 (24.0 – 47.0) | 8 | 34.0 (13.0 – 61.0) | 0.65 |
| Sperm progression (%)^#^ | 3 | 2.5 (2.0 – 14.0) | 3 | 32.0 (19.0 – 39.0) | 0.10 |
| Sperm normal morphology (%)^#^ | 5 | 4.0 (1.0 – 10.0) | 8 | 2.5 (2.0 – 6.0) | 0.65 |
| Age at achievement of maximal TV^#^ | 5 | 18.5 (15.2 – 20.0) | 12 | 19.2 (17.1 – 21.9) | 0.38 |
| Increase in TV after therapy (mL)^#^ | 5 | 9.0 (6.0 – 11.0) | 12 | 10.5 (2.0 – 22.0) | 0.20 |
| Maximal TV achieved (mL)^#^ | 5 | 12.0 (8.0 – 12.0) | 12 | 15.0 (12.0 – 25.0) | 0.001 |
| Increase in serum FSH after therapy (IU/L)^Ω^ | 8 | 5.1 (1.9 – 5.7) | 9 | 3.2 (1.8 – 5.1) | 0.14 |
| Increase in serum inhibin B after therapy (pg/mL)^Ω^ | 5 | 68.1 (62.6 – 84.7) | 12 | 65.9 (-64.8 – 161.3) | 0.78 |
| Change in serum AMH after therapy (pmol/L) ^Ω^ | 1 | -53.7 (-53.7) | 9 | -150.9 (-287.7 - -41.1) | N.A. |
| Increase in serum testosterone after therapy (nmol/L) ^Ω^ | 6 | 25.9 (6.9 - 28.5) | 11 | 25.1 (10.8 – 41.4) | 0.76 |

#Data are presented as median and range.

ΩData are presented as median and 95% confidence interval.

P values represent the statistical significance after performing a Mann-Whitney test between patients with Kallmann syndrome and those with non-Kallmann syndrome.

KS: Kallmann syndrome; normosmic CHH: congenital hypogonadotropic hypogonadism; yr: years: mo: months; IU/w: International Units per week; rFSH: recombinant follicle-stimulating hormone; hCG: human chorionic gonadotropin; TV: testicular volume; FSH: Follicle stimulating hormone; AMH: anti-Müllerian hormone; N.A.: Not assessed. Supplementary Table S4. Age and sex-matched reference values for serum concentrations of FSH, LH, testosterone and inhibin B, and age and Tanner stage-matched reference values for serum concentration of AMH.

| **Grinspon et al. 2012** | | | | |
| --- | --- | --- | --- | --- |
|  | **Age (yr.)** | **Median** | **3^rd^ – 97^th^ centile** |  |
| FSH (IU/L) | 15.0 – 17.9 | 3.21 | 1.4 – 8.5 |  |
| LH (IU/L) |  | 3.17 | 1.81 - 6.31 |  |
| **Bergadá et al. 1999** | | | | |
|  | **Age (yr.)** | **Mean** | **SEM** | **-2 SD – 2SD^#^** |
| T (nmol/L) | 12.0 - 19.0 | 11.3 | 1.9 | 7.5 – 15.7 |
| Inhibin B (pg/mL) |  | 400.0 | 70.2 | 259.6 – 540.4 |
| **Grinspon et al. 2011** | | | | |
|  | **Age (yr.) + G1** | **Median** | **3^rd^ – 97^th^ centile** |  |
| AMH (pmol/L) | 9.0 – 18.0 | 741.0 | 257 - 1371 |  |

#The values presented in this column were calculated based on the SEM values reported by Bergadá et al. 1999.

FSH: Follicle stimulating hormone; LH: Luteinising hormone; T: Testosterone; AMH: Anti-Müllerian hormone; SEM: Standard error of the mean; Yr.: year; G1: Tanner stage 1.
