# Supplementary material for: Effect of pubertal induction with combined gonadotropin therapy on testes development and spermatogenesis in males with gonadotropin deficiency: a cohort study
Source: Hum Reprod Open. 2025 May 13;2025(2):hoaf026. doi: 10.1093/hropen/hoaf026 (PMC12132099; doi:10.1093/hropen/hoaf026)
Supplement: hoaf026_Supplementary_Data [file hoaf026_supplementary_data.zip › Supplementary File S1.docx]

**Protocol for Induction of Puberty with Gonadotropins in Adolescent and Young Adult Males with GnRH or Gonadotropin Deficiency**

Authors: L Dunkel^1,2^, R Prasad,^1,2^, S Senniappan^3^, G Butler^4,5^, SR Howard^1,2^

^1^Centre for Endocrinology, Queen Mary University of London; ^2^Department of Paediatric Endocrinology, Barts Health NHS Trust; ^3^Department of Paediatric Endocrinology, Alder Hey Children’s NHS Foundation Trust; ^4^Department of Paediatric and Adolescent Endocrinology, University College London Hospital NHS Foundation Trust; ^5^UCL GOS Institute of Child Health, University College London

**Inclusion criteria:** Male patient ≥ 12 yrs of age *with*:

1. High clinical suspicion of hypogonadotropic hypogonadism by clinical and biochemical criteria (low or undetectable basal LH and FSH with low or undetectable testosterone), including *at least one of*
   1. Inhibin B <150pg/ml
   2. Red flags – cryptorchidism, micropenis, synkinesis, anosmia etc.
   3. Suspicion of evolving hypogonadotropic hypogonadism with additional combined pituitary hormone defects (GH, TSH, ACTH deficiency) (CPHD)
   4. MRI pituitary/ olfactory bulbs consistent with CHH or CPHD

*Or*

1. Diagnosis of hypogonadotropic hypogonadism or CPHD confirmed by genetics

*Or*

1. Diagnosis of hypogonadotropic hypogonadism by clinical and biochemical criteria (low or undetectable basal LH and FSH with low or undetectable testosterone) following history of acquired pituitary +/- hypothalamic damage e.g. due to tumour

*And*

Patient requiring treatment to induce or complete induction of puberty/ pubertal testicular maturation.

**Treatment protocol is divided into 2 protocol options**

1. For Patients with baseline Testes Volume < 4ml i.e. more severely affected patients who have signs of lack of gonadotropin stimulation during mini-puberty. These patients receive rFSH “pre-treatment” prior to adding in hCG.
2. For Patients with baseline Testes Volume ≥ 4ml i.e. less severely affected patients who have had some previous gonadotropin stimulation and receive hCG +/- rFSH.

***Therapy* and Monitoring in*** ***Patients with Testes Volume < 4ml***

*Baseline investigations:* serum (S)-LH, S-FSH, S-testosterone, S-E2, S-inhibin B, Testis volume (US), Tanner stages and bone age (BA) if not done in the last 6 months.

1. rFSH 150 IU sc three times per week on Mon/Weds/Fri for 2-4 months

Close monitoring^ of serum FSH to ensure target levels (4-6 IU/l)

rFSH dose adjustment as needed (if serum FSH < 4 IU/L, increase incrementally up to 225 IU every other day)

After a minimum of 2 months with serum FSH in target range, repeat the baseline investigations including testis US and start:

1. hCG 500-1000IU sc once weekly, while continuing rFSH 150-225 IU sc three-four times weekly (to keep FSH 4-6IU/L)

*Ongoing assessment and investigations:* Measure peak^^ S-testosterone 3-4 weeks after starting hCG therapy. Monitoring of Tanner stages, presence of gynaecomastia, testis volume, peak S-testosterone, S-E2, S-FSH, S-inhibin B and testis volumes every 3-6 months for the first 18 months. BA annually.

Individual responses to hCG vary. Dose adjustment of hCG may be needed to achieve peak testosterone (serum) in the low normal adult range (around 10nmol/L) after 6 months, and 20nmol/L after one year of start of hCG (after two years in patients <14 yrs of age)

If required, hCG dose can be increased in a stepwise fashion, to 1000IU once weekly, then to 1000IU twice weekly, and then to a maximum of 1,500 IU twice weekly.

*Semen analysis:* once patient is able to produce a semen sample and has attained testicular volume >10-12ml. Early morning urine sample for spermaturia can also be checked depending on local protocols. The timeframe from start of induction to semen analysis is variable but should be completed within 3 years.

*Training and support for sub-cutaneous injections provided by endocrine CNS team.

^Monitoring of FSH initially at 14 days post starting treatment, then repeated 10-14 days after dose adjustment or at 4-6 weeks post starting treatment if no dose adjustment.

Samples to be taken pre dose.

^^Measure peak s-testosterone by taking sample 3-4 days after the hCG injection.

***Therapy* and Monitoring in Patients with Testes Volume ≥ 4ml^†^***

*Baseline investigations:* S-LH, S-FSH, S-testosterone, S-E2, S-inhibin B, Testis volume (US), Tanner stages and BA if not done in the last 6 months.

1. hCG 500-1,000 IU sc once/twice weekly

Advise to start with the lower end of the dose range in younger patients, especially ≤13 yrs. Titrate dose based on peak^^ S-testosterone in the low normal adult range (around 10 nmol/L).

*Ongoing assessment and investigations:* Measure peak^^ S-testosterone 3-4 weeks after starting hCG therapy. Monitoring of Tanner stages, presence of gynaecomastia, testis volume, peak^^ S-testosterone, S-E2, S-FSH, S-inhibin B and testis volumes every 3-4 months for the first 18 months. BA annually.

*Semen analysis:* once patient is able to produce a semen sample and has attained testicular volume > 10-12ml. *If no sperm production on semen analysis, no further increase in testes volume or falling inhibin B by 6-12 months change to:*

1. rFSH 150 IU sc three times per week **+** hCG 1,000 IU sc once/twice weekly

Close monitoring of S-FSH^ to ensure target levels (4-6 IU/l) with rFSH dose adjustment as needed (if S-FSH < 4 IU/L, increase incrementally up to 225 IU every other day)

Close serial monitoring of peak^^ S-testosterone.

*Ongoing assessment and investigations:* Monitoring of Tanner stages, presence of gynaecomastia, testis volume, peak S-testosterone, S-E2, S-FSH, S-inhibin B and testis volumes every 3-4 months for the first 18 months. BA annually.

Individual responses to hCG vary. Dose adjustment of hCG to achieve peak testosterone (serum) in the low normal adult range (around 10nmol/L) after 6 months and 20nmol/L after one year of start of hCG.

If required, hCG dose can be increased to twice weekly up to 1,500 IU/dose.

Consider hCG dose reduction if gynaecomastia or excessive acne occur

*Semen analysis:* once patient is able to produce a semen sample and has attained testicular volume > 10-12ml

*Training and support for sub-cutaneous injections provided by endocrine CNS team.

^Monitoring of FSH initially at 14 days post starting treatment, then repeated 10-14 days after dose adjustment or at 4-6 weeks post starting treatment if no dose adjustment. Samples to be taken pre dose.

^^Measure peak s-testosterone by taking sample 3-4 days after the hCG injection.

***Special considerations***

1. Testosterone naïve patients or those with early arrested puberty

- Lower starting dose of hCG to 500 IU sc once per week for first 6 months of treatment
- Aim for peak testosterone (serum) around 5 nmol/L in the first 6 months of treatment
- Increase to 1000 IU once/twice per week for 2^nd^ 6 months of treatment with aim for peak serum testosterone of 10 nmol/L

1. Testosterone pre-treatment

- Testosterone pre-treatment is not a barrier to use of gonadotropins
- Testosterone therapy can be continued (or commenced) during the use of rFSH to maintain (or elevate) testosterone concentrations, and then discontinued when hCG is introduced
- Close monitoring as per the protocol above should be observed

1. Patients with hypopituitarism or reduced adult height prediction:

- In addition to annual BA, carry out annual calculation of delta BA / delta CA ratio and adult height predictions to monitor growth through puberty

***^†^***In patients with testes volumes of ≥4mls it is assumed that Sertoli cell proliferation during mini-puberty has occurred and that therefore these patients do not require FSH therapy to induce puberty. However, in some patients with testes volumes ≥4mls there may still be concerns, due to e.g. low inhibin B concentrations, about Sertoli cell number. In this case treatment with rFSH is advised to start alongside hCG therapy from the introduction of gonadotropin therapy.
